# Phase-specific postural adjustments in children and adults during a challenging balance task

**DOI:** 10.3389/fnint.2026.1734938

**Published:** 2026-02-17

**Authors:** Ludvík Valtr, Lucia Bizovská, Reza Abdollahipour, Bouwien Smits-Engelsman

**Affiliations:** 1 Department of Natural Sciences in Kinanthropology, Faculty of Physical Culture, Palacký University Olomouc, Olomouc, Czechia; 2 Physical Activity, Sport and Recreation, Faculty Health Sciences, North-West University, Potchefstroom, South Africa

**Keywords:** adults, anticipatory postural adjustments (APA), children, developmental differences, postural control

## Abstract

**Introduction:**

Anticipatory postural adjustments (APAs) stabilize the body before voluntary movement. Although present early in life, their refinement continues into adolescence, especially during complex balance tasks.

**Aim:**

This study examined developmental differences in APA control between typically developing children (9–12 years) and young adults (19–25 years) during a self-initiated Can Placement Task (CPT).

**Methods:**

Thirty children and twenty-two adults performed the CPT while standing on one leg. The task was divided into five phases (quiet stance, stooping, can transfer, straightening up, stabilization). Center of pressure (COP) displacement and velocity in anteroposterior (AP) and mediolateral (ML) directions and vertical ground reaction force (GRF) on the can were measured using dual force platforms. Both discrete outcomes and Statistical Parametric Mapping (SPM) were analyzed.

**Results:**

No differences were observed in static balance (Phase I). In dynamic phases, adults showed larger backward COP shifts during stooping, higher normalized COP velocity, and reduced reliance on the can for support compared with children. Children exhibited slower COP adjustments and higher GRF on the can, indicating greater use of external support. SPM revealed group differences mainly during stooping and straightening phases. Adults’ faster COP control likely reflects efficient feedforward strategies, while children adopted more conservative, stability-oriented approaches.

**Conclusion:**

Children aged 9–12 years can generate APAs but remain less efficient in adapting them to task demands. Phase-specific and SPM analyses revealed subtle developmental differences not evident in static balance. The CPT provides a sensitive framework for assessing postural control and may guide age-appropriate clinical interventions.

## Introduction

Maintaining postural stability is a fundamental requirement for nearly all voluntary movements, serving as the neuromuscular foundation for coordinated and purposeful action. To maintain upright balance and body alignment, the central nervous system (CNS) integrates visual, vestibular, and somatosensory inputs with motor commands that regulate postural control (Takakusaki, 2017). A key component of this control system involves anticipatory postural adjustments (APAs), which are feedforward mechanisms that prepare the body in advance of voluntary or predictable perturbations (Santos et al., 2010b). APAs are unconscious, feedforward muscular activations generated by the CNS to counteract the destabilizing effects of impending voluntary movements (Aruin and Latash, 1995; Bouisset and Do, 2008). They precede the primary action and reflect the brain’s ability to predict and minimize postural disturbances, thereby reducing reliance on reactive mechanisms. APAs serve to limit postural displacement and reduce the need for reactive, feedback-driven corrections, which occur after the perturbation and are typically less efficient (Alexandrov et al., 2005). It has been demonstrated that when perturbations are predictable, individuals who generate stronger APAs require smaller compensatory responses, leading to reduced center of pressure (COP) displacements and velocity and more stable posture (Santos et al., 2010a, b). Thus, the ability to generate timely and well-coordinated APAs is a critical indicator of efficient motor control. However, despite evidence for the prolonged developmental trajectory of APAs, no study to date has systematically compared APA dynamics across the full movement sequence (i.e., from initiation to completion) between older children and young adults, particularly in tasks that involve substantial internal perturbations such as single leg reaching. This gap limits our understanding of how anticipatory control mechanisms evolve in more ecologically valid and posturally demanding contexts.

Although the role of APAs in postural control is well established, their developmental progression–especially in tasks with greater postural complexity–remains incompletely understood. This is particularly relevant given the evidence that APA development is both prolonged and context-sensitive, with maturation varying across spatial and temporal domains. Understanding this developmental trajectory is essential for identifying age-related changes in motor control and balance. Research has shown that while children as young as 3–4 years demonstrate the presence of APAs (Mani et al., 2021), the refinement of these mechanisms continues into late childhood and adolescence (Barlaam et al., 2012; Girolami et al., 2010; Mani et al., 2019; Schmitz et al., 2002). For instance, spatial aspects of APAs, such as the amplitude of COP shifts, reach adult-like levels between 7 and 10 years of age during relatively simple tasks like arm movements or single-leg standing (Girolami et al., 2010; Hay and Redon, 2001; Mani et al., 2019). However, during dynamic tasks, the development of temporal control and intersegmental coordination continues to mature well into adolescence, suggesting that the neural circuits underlying precise timing and multi-joint motor planning remain under development beyond childhood (Barlaam et al., 2012; Girolami et al., 2010; Mani et al., 2019).

In their gait initiation study, Mani et al. (2021) demonstrated direction-specific developmental trends, showing that mediolateral (ML) APA peak amplitudes increased until approximately 7–8 years of age, then gradually decreased and approached adult levels by around age 10, while temporal parameters and coordination patterns, such as COP vector trajectory, remained immature even at that age. These findings support the notion that APAs development is multifactorial and protracted, involving distinct neural substrates that mature asynchronously (Cignetti et al., 2018; Jacobs et al., 2009). Moreover, these maturational differences are likely influenced by postural demands, task complexity, and movement context (Hadders-Algra, 2010; Winter et al., 1996). Specifically, ML control, which requires fine-tuned hip musculature and coordination for weight transfer, appears to develop later than anteroposterior (AP) control dominated by ankle musculature, which is recruited in more common daily activities (e.g., reaching, stepping). Thus, there remains a critical gap in the literature regarding direct comparisons between older typically developing children and young adults – particularly in tasks that impose high postural demands, such as standing on one leg while executing a self-induced reach. These tasks demand not only the generation of precise APAs but also the ability to maintain balance as the center of mass shifts dynamically–challenges that may expose subtle developmental immaturities not detectable in easier movements. Thus, a systematic comparison of APA characteristics between typically developing children and young adults using a challenging, internally perturbed balance task is warranted.

Many studies have focused solely on the brief period preceding movement onset, traditionally associated with APAs (Duarte et al., 2022). However, to fully capture the demands of postural control and the strategies used to manage them, it is essential to analyze postural behavior across the entire movement sequence–from initiation to completion. Postural control is not limited to the preparatory phase; when anticipatory mechanisms are suboptimal, individuals often rely on compensatory strategies during execution and recovery phases. These responses can be observed through greater overall COP path lengths and increased COP velocities, which indicate reduced stability and less efficient feedforward control (Valtr et al., 2024). Analyses restricted to the initial APA period may therefore underestimate the complexity and demands of postural regulation, especially in dynamic and ecologically valid tasks. To address this, our study employed the Can Placement Task (CPT), a single-leg balance task designed to elicit self-induced perturbations and challenge postural stability. The task consists of five distinct phases: Phase I involves a quiet single-leg stance prior to movement, during which no major differences between children and adults are expected due to its static nature. Phases II, III, and IV include reaching for the can, transporting it, and returning to upright stance–each involving dynamic shifts in posture that require effective anticipatory postural control. Finally, Phase V represents the post-movement stabilization phase, where participants regain steady single-leg stance. The CPT was designed to challenge dynamic stability, as stooping and placement can induce large COP shifts and may lead to loss of balance if not performed under finely tuned anticipatory control. During forward trunk motion, the body geometry changes, and the center of gravity is displaced. Through repeated experience, performers learn to anticipate expected changes and to generate anticipatory forces opposite to the reaction forces associated with trunk movement, thereby minimizing postural disturbance. This approach applies a detailed, phase-specific analysis of COP behavior across task segments, revealing how postural control in the CPT emerges from a dynamic interplay between anticipatory and compensatory mechanisms (Smits-Engelsman et al., 2020; Valtr et al., 2024). Phase II relies mainly on anticipatory postural adjustments, because predicting self-induced perturbation enables stabilization by bypassing perceptual delays. Phase III is likely influenced by compensatory mechanisms, while still reflecting downstream consequences of the preceding APA, in that more effective anticipatory organization may reduce the need for corrective actions and decrease reliance on external support, quantified here by smaller vertical force on the can. Phases IV and V add recovery demands during the return to upright stance and subsequent stabilization, increasing the need for corrective control when the anticipatory set is insufficient. Comparing typically developing children with healthy adults provides a window into developmental constraints: adults exhibit fully matured feedforward control and efficient postural strategies, while children reveal how postural control evolves as predictive mechanisms and compensatory responses mature. Tasks involving reaching, transporting and stabilizing objects are particularly informative because they impose predictable perturbations and highlight the sequential involvement of postural control mechanisms. Phase-specific analysis allows for the identification of where control is most challenged, during initiation, disturbance management, or recovery–rather than reducing balance to a single global measure. This approach clarifies how anticipatory and compensatory strategies develop, illustrating the gradual shift from feedback-dominated to feedforward-dominated postural control.

To further enhance the sensitivity and resolution of our analysis, we employed Statistical Parametric Mapping (SPM) to assess continuous changes in COP displacement and velocity throughout the entire CPT. Traditional discrete-point analyses, such as comparing peak values or onset latencies, often oversimplify the complex and time-varying nature of postural control (Pataky et al., 2016). In contrast, SPM allows for a statistically robust, point-by-point comparison of time-series data across the full duration of the movement, allowing us to detect precisely when significant group differences emerge. This approach is particularly valuable in developmental studies, where anticipatory and compensatory postural strategies may vary subtly over time. For example, two age groups may show similar COP peak values, yet differ in the timing, duration, or variability of their postural adjustments–differences that discrete measures may fail to detect. By using SPM, we can capture these temporal dynamics in a continuous fashion and relate them directly to the functional integrity of feedforward motor control. Moreover, this method allows us to evaluate anticipatory postural adjustments across the entire task duration, rather than at isolated time points, to fully understand their developmental progression.

Therefore, the aim of the present study was to compare anticipatory postural adjustments in typically developing children and young adults during a self-initiated CPT performed while standing on one leg. We hypothesized that developmental differences in APA control would vary across movement phases, with children showing greater COP instability–particularly during the more demanding phases involving stooping, can transfer, and recovery–reflecting increased reliance on reactive postural strategies. We also expected that these group differences would be most evident in measures of COP velocity during dynamic segments of the task, such as stooping and standing up, rather than during more stable stance phases. Finally, we anticipated that adults would exhibit reduced reliance on external support, indicated by lower maximum vertical ground reaction force (GRF) on the can.

## Methods

### Participants

Thirty typically developing children aged between 9 and 12 years and twenty-two young adults between 19 and 25 years participated in the study. *A priori* power analysis with G*Power 3.1 indicated that sample size of *n* = 20 per group would be sufficient to identify significant differences between groups in a within-between design with a power (1−β) of 0.95, a medium effect size *f* of 0.24, the number of measurements = 4, correlation among repeated measures = 0.50, non-sphericity correction ε = 1, and an α level of 0.05 (Faul et al., 2007). Demographic and clinical descriptions of the groups are shown in Table 1. All participants were free from neurological, musculoskeletal, or developmental disorders. In children, typical motor development was confirmed through scores above the 16th percentile on the Movement Assessment Battery for Children – Second Edition (MABC-2) and absence of relevant diagnoses in school-provided anamnesis. For adults, typical development and health status were confirmed via self-report. Children were recruited through local elementary schools, and young adults were recruited from a university student population. The study sample reflects the demographic composition of the regional population (predominantly White/Central European). Information about participants’ race/ethnicity and socioeconomic status was not systematically collected, as these data are not routinely gathered in the national educational or institutional recruitment contexts from which participants were drawn. Written informed consent was obtained from adult participants and from the parents or guardians of all children, with assent provided by the children themselves. The study protocol was approved by the institutional ethics committee (Ethical Committee of the Faculty of Physical Culture, Palacký University Olomouc, FTK 46/2020) and adhered to the principles of the Declaration of Helsinki.

**TABLE 1:** Participants data (presented as a mean ± standard deviation).

Participant characteristics	Children	Adults
*n* participants (males)	30 (15)	22 (11)
Age (years)	10.2 ± 1	21.8 ± 1.5
Body mass (kg)	39.4 ± 10.4	71 ± 12.1
Height (m)	1.47 ± 0.08	1.76 ± 0.11
MABC-2 percentile	63%	–
MABC-2 balance sum score	30.5 ± 2.4	–
Additional trials CPT (#)	1.6 ± 1.9	0.2 ± 0.5

CPT, Can Placement Task; #, number.

### Experimental procedure

**FIGURE 1:**
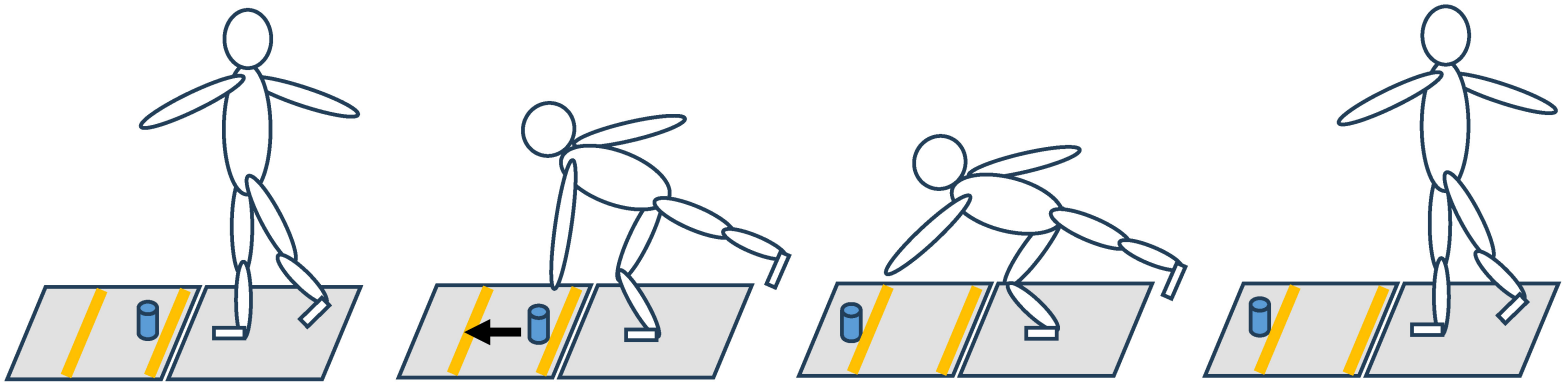
Schematic of modified Can Placement Task.

The experimental procedure was identical to that used in Valtr et al. (2024), employing a modified version of the CPT derived from the PERF-FIT test battery (Figure 1). In this adaptation, a single can was moved during each trial, rather than four as in the original clinical version. Participants were instructed according to the standardized PERF-FIT manual. They were shown how to stand upright on one leg with the toes of the supporting foot close to the first yellow line, then bend forward, pick up one can, and place it close behind second line before returning to an upright position while maintaining single-leg stance. The emphasis was on correct execution and accurate placement of the can while avoiding leaning on the can for support. No explicit instruction regarding movement speed was given, and participants performed the task at a self-selected comfortable pace, focusing on completing the movement correctly while maintaining balance. They were also told that the non-supporting leg should remain free but was not allowed to touch the ground or the stance leg during the trial. Throughout the task, the upper limbs were kept relaxed and allowed to move naturally. Each trial consisted of five distinct phases: (I) initial quiet stance (fixed time interval 3 s), (II) stooping to pick up the can from a low platform, (III) transporting the can through the air without contact with the force platform, (IV) placing the can down and straightening up, and (V) final quiet stance (fixed time interval 3 s). For precise detection of phases of movement, participants stood on one platform, a can was placed on the other one (Figure 1). Five abovementioned phases of movement were recognized based on the behavior of the vertical component of GRF recorded with the platform a can was placed on. Phase III was identified as a period during which a force value less than 0 N was observed. Two task conditions were used. In the “further” condition, the can was initially placed in front of the standing line and participants were instructed to move it forward beyond a second line positioned 35 cm ahead. In the “closer” condition, the can started beyond the second line and was moved backward to a position just in front of the standing line. These conditions replicate the standardized setup of the CPT as defined in the PERF-FIT test battery. Their inclusion ensures consistency with this validated clinical protocol and preserves ecological validity. Participants completed familiarization trials until they were able to perform the task correctly with each leg. Then, three successful repetitions of each can movement type (closer and further) were performed on each supporting leg (left and right), resulting in a total of 12 trials per participant. Only successful trials were included in the analysis. The number of additional (error) trials required to complete all 12 repetitions is presented in Table 1. The order of supporting leg was randomized to minimize order effects, whereas the order of task conditions (closer vs. further) was kept fixed to remain consistent with the standardized PERF-FIT protocol.

### Data acquisition and processing

Two force platforms (AMTI OR6-5, Advanced Mechanical Technology, Inc., Watertown, MA, USA, sampling rate 200 Hz) were used for the recording of the GRF and COP movement. The vertical component of GRF and COP position data were filtered using a 4th order bidirectional low-pass Butterworth filter with a cut-off frequency of 10 Hz. All data together with instantaneous COP velocities computed after filtering were divided into the five phases of the CPT (Valtr et al., 2024) and duration of phases II, III, and IV was computed. Each trial was then time-normalized to a uniform number of data points for every person and can movement condition. Considering the approximate differences of length of phases of movement, the time normalization was performed using the “pchip” interpolation type with the final data points number of 600 (3 s as per the original 200 Hz sampling rate) for phases I, II, IV and V and 200 for phase III resulting in 2600 total samples (13 s) in total for analysis of the whole time series. To ensure that the data on can movement distance (fixed at 35 cm) were independent of participants’ body height, the time series of COP position data in each direction were normalized using the following index before further processing: Index = (can movement distance/participant height) × 100. This ensures movement distance is interpreted relative to body size. To remove baseline offsets in COP position, detrending was applied using the mean COP position value in each direction during phase I. GRF values were normalized to participants’ body weight. For each participant and can movement condition, median time series was computed for COP positions and COP velocities in AP and ML directions. These time series were used for statistical parametric mapping procedures as described below. Moreover, from these time series, discrete values corresponding to total COP path length, range of motion and mean velocities in AP and ML directions were computed for each phase of movement. Maximum relative vertical GRF during phases II and IV were also included.

### Statistical analysis

All data processing was performed in Matlab (R2022b, MathWorks, Inc., Natick, MA, USA). Statistical analysis for continuous data was performed by algorithms available on (version M.0.4.10, Pataky, 2012) with 2-way mixed Analysis of variance (ANOVA) with groups (children vs. adults) as a between-subject factor and conditions (farther vs. closer) as within-subjects factors. Effect size values for ANOVA were quantified using partial eta squared (η^2^), where η^2^ = 0.01, 0.06, and 0.14 corresponded to a small, moderate, and large effect, respectively (Cohen, 2013). To estimate the effect sizes for *post hoc* comparisons Cohen’s d was utilized. The evaluation of Cohen’s d corresponded to a low (*d* = 0.2), medium (*d* = 0.5), and large (*d* = 0.8) effect (Cohen, 2013). The significance level was set at 0.05. Discrete data were statistically processed using IBM SPSS Statistics (v. 26, IBM, NY, USA) with similar settings, however, taking into consideration the phase of movement. Three-way mixed ANOVA with groups (children vs. adults) as a between-subject factor and conditions (farther vs. closer) and phase of movement (3 for phase duration, 5 for COP data, 2 for GRF data) as within-subjects factors. As in previous research (Valtr et al., 2024), a preliminary analysis–conducted but not included in this manuscript–was performed using a mixed ANOVA with gender and leg as between-subjects factors. This analysis revealed no significant main effects or interactions involving either gender or leg. Therefore, these results are not reported here. For the analyses presented in this study, average values across both lower limbs were used. Kolmogorov-Smirnov test was used for confirmation of normal data distribution beforehand and the Bonferroni *post-hoc* test was applied for pairwise comparisons.

## Results

### Mixed-design ANOVA results

Descriptive data and the results of factorial ANOVA are described in Table 2.

**TABLE 2:** Descriptive statistics (presented as a mean ± standard deviation) and results of mixed ANOVA of the force plate variables in the closer and further condition.

Variable/ phase	Closer		Further		ANOVA	
	Adults	Children	Adults	Children	Effect	*p*
**Phase duration (s)**						
II	2.51 ± 0.48	3.20 ± 0.64	2.57 ± 0.48	2.87 ± 0.51	G	<0.001
III	0.99 ± 0.20	1.35 ± 0.32	1.05 ± 0.21	1.26 ± 0.31	C	**0.022**
IV	2.73 ± 0.32	3.98 ± 0.61	2.71 ± 0.26	3.94 ± 0.44	P	**<0.001**
					G*C*P	**0.006**
**Path length (mm %^–1^)**						
I	4.74 ± 1.17	4.87 ± 1.20	4.78 ± 1.02	5.05 ± 1.07	G	0.471
II	9.27 ± 2.81	9.45 ± 2.74	8.82 ± 3.21	8.17 ± 2.00	C	0.314
III	3.85 ± 0.82	4.64 ± 1.20	4.24 ± 1.20	4.70 ± 0.94	P	**<0.001**
IV	9.63 ± 2.22	10.94 ± 2.86	10.72 ± 2.82	11.83 ± 2.58	G*C*P	0.411
V	4.92 ± 1.30	4.59 ± 1.43	5.18 ± 1.23	4.66 ± 1.41		
**Mean velocity AP (mm s^–1^%^–1^)**						
I	1.13 ± 0.24	1.21 ± 0.30	1.18 ± 0.25	1.24 ± 0.27	G	**0.001**
II	2.94 ± 0.54	2.45 ± 0.54	2.80 ± 0.51	2.40 ± 0.45	C	**<0.001**
III	3.11 ± 0.71	2.51 ± 0.47	3.25 ± 0.71	2.99 ± 0.68	P	**<0.001**
IV	2.89 ± 0.57	2.21 ± 0.37	3.10 ± 0.61	2.37 ± 0.37	G*C*P	**0.013**
V	1.27 ± 0.29	1.14 ± 0.29	1.35 ± 0.31	1.17 ± 0.28		
**Mean velocity ML (mm s^–1^%^–1^)**						
I	1.13 ± 0.24	1.10 ± 0.24	1.13 ± 0.24	1.16 ± 0.30	G	**<0.001**
II	2.34 ± 0.55	1.83 ± 0.38	2.22 ± 0.53	1.88 ± 0.38	C	**0.029**
III	2.89 ± 0.78	2.36 ± 0.49	2.91 ± 0.80	2.60 ± 0.64	P	**<0.001**
IV	2.57 ± 0.56	1.82 ± 0.30	2.64 ± 0.56	1.97 ± 0.34	G*C*P	0.071
V	1.18 ± 0.26	1.02 ± 0.22	1.30 ± 0.33	1.02 ± 0.26		
**Range AP (mm %^–1^)**						
I	0.70 ± 0.20	0.66 ± 0.24	0.72 ± 0.25	0.68 ± 0.16	G	0.707
II	2.85 ± 0.86	2.85 ± 0.57	1.90 ± 0.86	1.95 ± 0.6	C	0.297
III	1.50 ± 0.60	1.55 ± 0.46	1.54 ± 0.50	1.71 ± 0.4	P	**<0.001**
IV	1.52 ± 0.52	1.57 ± 0.45	2.33 ± 0.91	2.81 ± 0.62	G*C*P	0.116
V	0.94 ± 0.48	0.81 ± 0.44	0.93 ± 0.30	0.67 ± 0.27		
**Range ML (mm %^–1^)**						
I	0.68 ± 0.21	0.62 ± 0.21	0.61 ± 0.18	0.67 ± 0.17	G	0.164
II	0.97 ± 0.29	0.88 ± 0.29	0.93 ± 0.31	0.89 ± 0.20	C	0.270
III	0.74 ± 0.25	0.80 ± 0.23	0.88 ± 0.29	0.77 ± 0.18	P	**<0.001**
IV	1.16 ± 0.38	0.99 ± 0.27	1.24 ± 0.31	1.03 ± 0.33	G*C*P	0.114
V	0.68 ± 0.17	0.62 ± 0.22	0.66 ± 0.18	0.70 ± 0.42		
**Max GRF (% body weight)**						
II	0.26 ± 0.11	0.56 ± 0.37	0.38 ± 0.29	0.77 ± 0.76	G	**<0.001**
IV	0.27 ± 0.14	0.49 ± 0.39	0.19 ± 0.11	0.47 ± 0.38	C	0.112
					P	**0.032**
					G*C*P	0.836

AP, anterioposterior; ML, mediolateral; GRF, ground reaction force; G, group; C, condition; P, phase; I, single-leg stance; II, stooping; III, can transfer; IV, straightening up; V, stabilization. Significant results with *p*-values smaller than 0.05 are in boldface.

### Group main effect

No significant group effect was observed for COP path length (*p* = 0.471), indicating that the total length of COP excursion during the task did not significantly differ between children (6.89 ± 3.32 mm %^–1^) and adults (6.61 ± 3.16 mm %^–1^). This global (aggregated) measure reflects the overall amount of COP travel accumulated across the entire task and therefore does not capture directional or phase-specific shifts in COP position. Significant group differences emerged for AP COP velocity [*F*(1, 52) = 13.984, *p* = 0.001, η^2^ = 0.191] and ML COP velocity [*F*(1, 52) = 16.109, *p* < 0.001, η^2^ = 0.220]. Adults demonstrated significantly higher AP velocity (2.3 ± 1.01 mm s^–1^ %^–1^) compared to children (1.97 ± 0.78 mm s^–1^ %^–1^). Similarly, adults exhibited significantly higher ML velocity (2.03 ± 0.88 mm s^–1^ %^–1^) than children (1.68 ± 0.66 mm s^–1^ %^–1^). Movement duration in Phases II–IV was longer in children than in adults [*F*(1, 52) = 73.929, *p* < 0.001, η^2^ = 0.597]. Taken together, these outcomes indicate comparable COP path length between groups, alongside significantly lower mean COP velocities in children than in adults in both the AP and ML directions. In addition to differences in COP velocity, a significant group effect was also observed for maximum GRF. Children (0.57% ± 0.51% body weight) showed significantly higher maximum vertical GRF on the can than adults (0.28% ± 0.19% body weight) during the phases involving contact with the can (stooping and straightening up).

### Interaction effects

A significant three-way interaction between group, can movement and phase was found for mean AP velocity [*F*(4, 52) = 2.759, *p* = 0.008, partial η^2^ = 0.459] and Phase duration [*F*(2, 52) = 5.348, *p* = 0.006, partial η^2^ = 0.097]. *Post hoc* analysis of Phase duration indicated that in adults, movement duration did not differ between conditions across phases, whereas in children, Phase II (stooping, *p* < 0.001, *d* = 0.587) and Phase III (can transfer, *p* = 0.012, *d* = 0.288) were longer in the closer condition than in the further condition. *Post hoc* analysis also showed that AP velocity was significantly higher in adults than in children across several phases and both movement conditions. Specifically, in the closer condition, significant group differences were found in Phase II (stooping, *p* = 0.002, *d* = 0.910), Phase III (can transfer, *p* = 0.001, *d* = 0.998), and Phase IV (straightening up, *p* < 0.001, *d* = 1.416). In the further condition significant group differences were also observed in Phase II (stooping, *p* = 0.005, *d* = 0.820), Phase IV (straightening up, *p* <0.001, *d* = 1.439), and Phase V (stabilization, *p* = 0.032, *d* = 0.611). Thus, the direction of the group effect was consistent across these phases, with higher mean AP velocity in adults than in children. Furthermore, in Phase II (stooping), adults exhibited significantly higher AP velocity when moving the can toward themselves (closer) compared to moving it away (further) (*p* = 0.016, *d* = 0.273). This difference was not observed in children (*p* = 0.300, *d* = 0.102) indicating that a within-group condition difference in Phase II was present in adults but not in children. In Phase III (can transfer), the pattern was reversed and observed only in children, who showed significantly higher AP velocity in the further condition compared to the closer condition (*p* < 0.001, *d* = 0.809). This effect was not present in adults (*p* = 0.181, *d* = 0.186). In Phase IV (straightening up), both groups demonstrated significantly higher AP velocity in the further condition (*p* = 0.006, *d* = 0.344 for adults, *p* = 0.013, *d* = 0.420 for children), indicating a consistent within-group condition effect (further > closer) in Phase IV for both groups. Overall, the interaction reflected phase-dependent group differences and phase-dependent condition effects, with the direction and presence of these effects varying across phases and between groups.

### Statistical parametric mapping

**FIGURE 2:**
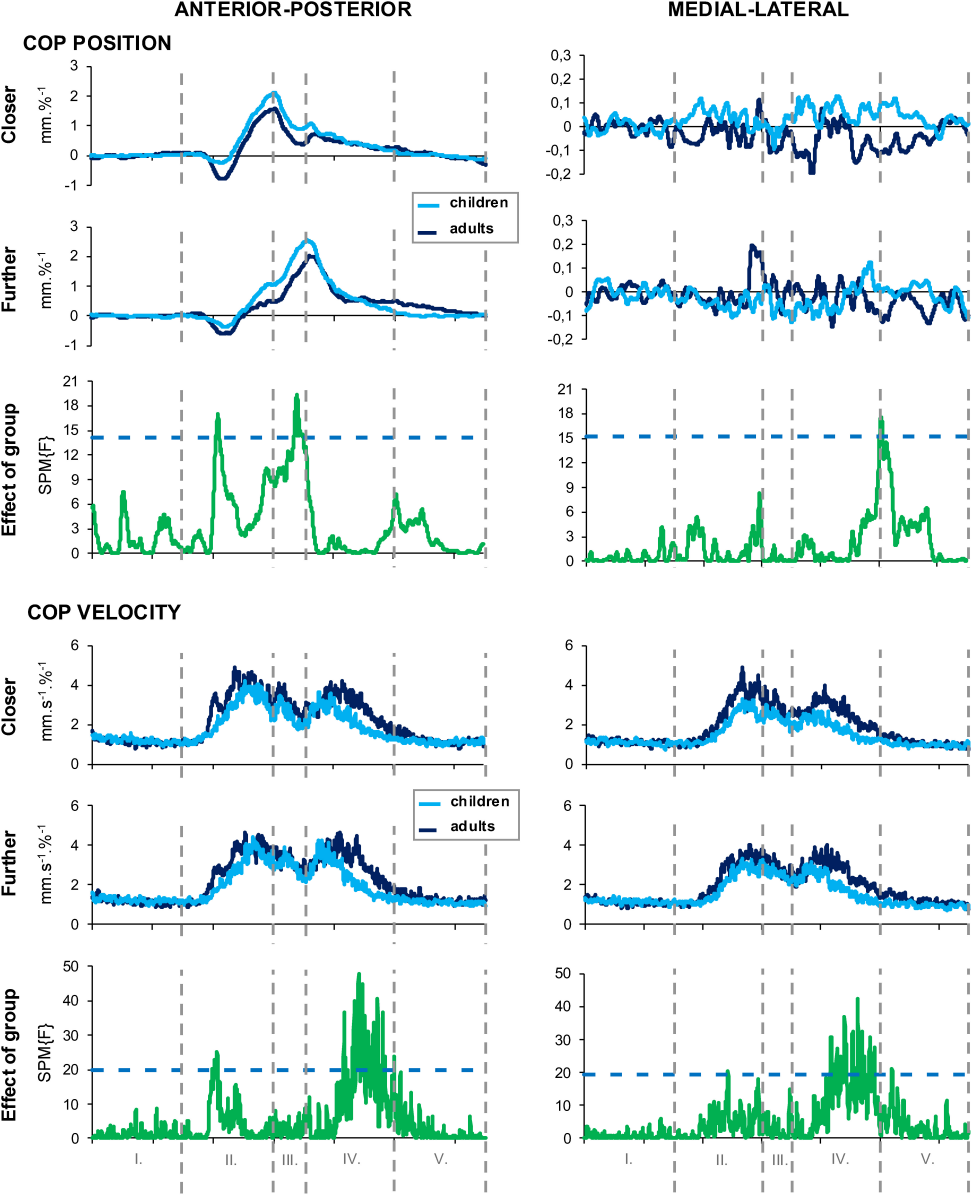
SMP analysis of COP dynamics across task phases. Time-series plots of COP position and velocity in the anterior-posterior (left panels) and medio-lateral (right panels) directions during the CPT. Data are shown separately for children (light blue) and adults (dark blue) under the closer and further can positions. The bottom panels show the results of SPM tests for group effects (green), with the dashed line indicating the critical threshold for significance (*p* < 0.05). Vertical dashed lines mark the five CPT phases: Phase I – single-leg stance, Phase II – stooping, Phase III – can transfer, Phase IV – straightening up, and Phase V – stabilization.

Statistical parametric mapping revealed distinct age-related differences in postural strategies, particularly in the AP dimension (Figure 2). During Phase II (stooping), adults exhibited a greater backward shift of the COP in the AP direction indicating a phase-specific directional change in COP position over time. This pattern represents a localized phase-specific change in COP position (and thus COP shift within Phase II), rather than a difference in the overall COP path length quantified by the aggregated ANOVA outcome. In contrast, during Phase III (can transfer), children showed a more pronounced forward lean, which was accompanied by greater reliance on the can for support as indexed by higher vertical GRF values. No significant group differences were found in the ML COP position across phases, suggesting comparable lateral stability. Regarding COP velocity, adults displayed higher AP velocity in Phase II, which corresponds with the backward shift in COP position and describes a concurrent phase-specific difference in the time course of AP COP velocity. In Phase IV (straightening up), both groups demonstrated a distinct pattern of rapid movement into the desired posture followed by fine corrective adjustments; however, children reached the target with lower velocity, while adults displayed more controlled and consistent corrections. Finally, ML COP velocity in Phase IV was significantly higher in adults, indicating more active lateral postural control during the transition to upright stance.

## Discussion

This study investigated developmental differences in APAs between TD children and young adults during a self-initiated balance task involving single-leg stance and a can placement movement. By segmenting the task into distinct movement phases and incorporating both COP metrics and GRF, we were able to characterize phase-specific postural control strategies and how they differ between typically developing children and young adults. No group differences were observed during Phase I, which represented static single-leg stance, suggesting that basic balance control was comparable between children and adults. Consistent with our hypotheses, children aged 9–12 years demonstrated lower COP velocity and greater reliance on external support during anticipatory postural control compared to adults. This was reflected in several key outcomes: (1) reduced COP velocity across both AP and ML axes, suggesting more cautious or constrained postural strategies; (2) significantly greater reliance on external support during the task, evidenced by increased vertical GRF exerted on the can; and (3) phase- and condition-specific variations in APA behavior that were less adaptive and more reactive in children.

The present results reinforce the view that APAs undergo a protracted developmental trajectory that extends into late childhood, particularly under complex and multi-phase task conditions. Previous developmental research suggests that although children achieve near-adult levels in the magnitude of their COP displacement relatively early, other aspects of APA control continue to evolve well beyond early childhood (Girolami et al., 2010; Hay and Redon, 2001). In particular, Mani et al. (2021) found that mediolateral APA amplitude tends to rise until around ages 7–8, after which it decreases, approaching the adult values by approximately age 10. However, the timing of APA initiation and the smooth coordination of multiple body segments do not follow this same trajectory, with both remaining less mature at this age. Our findings align with this pattern as children aged 9–12 years in the present study were able to perform the CPT but still demonstrated lower APAs efficiency than adults. This was evident in less consistent modulation of COP velocity during the stooping phase (Phase II), where adults adjusted velocity depending on can position, but children did not. In contrast, children exhibited increased AP velocity during the can transfer phase (Phase III) in the further condition, which may reflect later-emerging corrective adjustments during the transfer when phase-specific modulation is less consistent. In this context, the Phase III increase in AP velocity in the further condition may represent a compensatory response to maintain stability, consistent with the concurrently greater reliance on external support observed in children (i.e., higher vertical GRF applied to the can). Taken together, these phase- and condition-dependent patterns suggest that adults modulate anticipatory postural control more consistently in response to task constraints, whereas children show less consistent modulation, with condition effects emerging in later phases of the movement sequence. Importantly, this group-specific sensitivity to task constraints was also reflected in movement timing, with adults showing comparable phase durations across the closer and further conditions and children exhibiting longer Phase II (stooping) and Phase III (can transfer) durations in the closer condition. This suggests that condition effects in children extend beyond COP dynamics to the temporal organization of the action sequence. In addition, children exerted higher vertical ground reaction forces on the can throughout the task, indicating a greater tendency to lean on it for support. This reliance on external stabilization is consistent with the interpretation that immature APAs may be associated with greater use of compensatory upper limb support to maintain balance (Valtr et al., 2024). Such reliance likely reflects the need to compensate for less efficient anticipatory control, leading to reactive adjustments that can carry over into later movement phases, as previously observed in gait initiation and other dynamic balance tasks (Assaiante et al., 2000; Palluel et al., 2008). Collectively, these results support the view that while basic APA mechanisms are established by middle childhood, the fine-tuning of these responses and their flexible adaptation to varying task demands continue to mature into late childhood.

Phase-specific developmental differences in postural control have been observed in children compared with adults, particularly during tasks that require control of whole-body momentum. In both the sit-to-stand task (Suriyaamarit et al., 2022) and gait initiation task (Hirata et al., 2025), children exhibit greater COM displacement and velocity, especially in the early and momentum-transfer phases. Such patterns are generally interpreted in the postural control literature (Guetiti et al., 2024; Hirata et al., 2025) as signs of reduced efficiency, reflecting either excessive momentum generation or a greater reliance on corrective actions to maintain stability. While Suriyaamarit et al. (2022) observed these developmental differences in the sit-to-stand task, the exact nature of the differences was not identical across studies, highlighting task-specific characteristics of postural control development.

By contrast, in our CPT, adults–not children–showed a greater backward COP shift in the AP direction during Phase II (stooping). This larger anticipatory displacement in adults likely reflects a more appropriate feedforward strategy to position the center of mass in preparation for the reaching movement. Rather than indicating instability, such a shift is mechanically advantageous for counterbalancing the forward lean associated with stooping and therefore points to more proactive postural control (Lu et al., 2017; Stapley et al., 1998). Importantly, because the APA amplitude can be scaled by stability demands under more challenging conditions (Aruin et al., 1998), the smaller posterior COP shift in children may partly reflect task difficulty. However, this factor alone is unlikely to fully explain our findings. Children not only made a smaller shift but this co-occurred with greater reliance on external support, reflected by higher GRF on the can, and with condition-related modulation emerging later in the movement sequence. Together, these features are consistent with less mature feedforward organization rather than a uniform difficulty-driven attenuation.

When examining the entire task, COP path length was similar between adults and children, yet adults exhibited higher normalized COP velocity. Consistent with this, movement phases were completed faster by adults, with shorter durations in Phases II–IV compared with children. Because our COP velocity values were normalized for body size and reach distance, higher velocity in this context does not necessarily indicate instability. Instead, it likely reflects a more decisive and efficient execution of anticipatory postural adjustments. Adults appear able to reposition their COP more quickly to the desired location without increasing total displacement, consistent with a proactive, well-coordinated APA strategy. In contrast, children may employ slower, more tentative COP movements to preserve balance, indicative of less efficient anticipatory control. This interpretation is consistent with Hirata et al. (2025) who argued that children adopt a conservative, safety-oriented strategy rather than simply demonstrating inefficient postural control. Slower and less decisive COP shifts in children during the CPT may reflect a deliberate prioritization of stability over efficiency. Hirata et al. (2025) also emphasized that adults exploit dynamic efficiency by relying more on forward momentum and smaller safety margins in the AP direction, whereas children maintain larger safety reserves in the ML direction. Similarly, in our CPT, adults exhibited rapid and proactive COP adjustments, while children relied more on external support and slower weight shifts. Such differences suggest a broader developmental trend, with adults favoring efficient feedforward strategies and children adopting conservative, safety-first approaches given the task constraint of maintaining single-leg stance and avoiding falls. Consistent with this safety-first account, the phase-duration interaction showed stable timing in adults across conditions, whereas children spent longer in Phase II (stooping) and Phase III (can transfer) in the closer condition. This selective slowing aligns with the idea that children prioritize stability during the most demanding parts of the sequence.

One potential explanation for the higher COP velocity observed in adults relates to the ongoing maturation of the basal ganglia (BG). BG plays a central role in the sequential execution of complex movements (Jin and Costa, 2015; Yin, 2014), such as the successive components of our CPT (single-leg stance, stooping, reaching, grasping). In children aged 9–12 years, the BG are still undergoing structural and functional maturation (Wierenga et al., 2014). Insufficient maturation of these circuits may contribute to slower and less decisive sequencing of postural and reaching elements, thereby reducing COP velocity during task execution. A similar trend is observed in individuals with Parkinson’s disease, where BG dysfunction leads to slowing of movement sequences (Dalton et al., 2011; Jankovic, 2008). This parallel suggests that adults’ higher COP velocity may be related to more mature sequential motor control, whereas children’s slower COP adjustments may partly reflect developmental constraints in neural system. These behaviors can be understood as functional adaptations to ongoing neuromuscular development rather than as deficits (Hirata et al., 2025).

Additional factors are likely to contribute to children’s postural strategies. Compared to adults, children generally possess less muscular strength (Richardson et al., 2025) and greater variability in force production (Sosnoff et al., 2007), which may limit their ability to generate rapid COP shifts even when anticipatory strategies are engaged. Thus, the larger backward COP shift in adults during Phase II together with their higher normalized COP velocity across the whole task indicates a proactive approach to postural control, whereas children’s strategies reflect both developmental constraints and adaptive prioritization of stability. Importantly, the fine-tuning of anticipatory postural adjustments should not be viewed as a purely maturational process. Instead, it represents a learning trajectory that depends on accumulating experience across diverse motor contexts, where making and correcting errors gradually shapes more efficient feedforward control (Malone et al., 2025). This perspective highlights that the transition toward adult-like APA strategies emerges not only through neuromuscular development but also through practice-driven refinement of motor planning and postural coordination.

The present study has several important strengths and limitations. A key limitation lies in the age range of the child group, which represents a heterogeneous stage of development. Subtle age-related differences within this interval may therefore not have been fully captured. Another limitation is that the study employed a cross-sectional design, providing only a snapshot of developmental differences. This approach does not allow us to track the longitudinal progression of APA maturation within the same individuals, which would be valuable for understanding how postural control strategies evolve over time. Furthermore, the sample consisted of typically developing children and young adults from a relatively homogeneous Central European population. This demographic composition constrains the generalizability of the findings to similar sociocultural and educational contexts. Broader representation across diverse ethnic, socioeconomic, and cultural groups would be necessary to determine whether the observed developmental patterns reflect universal mechanisms of postural control or context-specific adaptations. An additional limitation is that the displacement of the can was fixed at 35 cm, as per the standardized PERF-FIT protocol, rather than being adjusted relative to participant height. While this approach ensures comparability with existing PERF-FIT data, it may have influenced task difficulty for individuals of different body sizes. To mitigate this, COP data were normalized during analysis; however, future studies should confirm these findings by adapting can displacement to a percentage of body height. Among the strengths, the novelty of the CPT represents a major contribution. Task complexity clearly influenced performance, revealing subtle developmental differences that might remain undetected in simpler balance tasks, which often fail to sufficiently challenge older children. In addition, the phase-specific analysis provided fine-grained insights into anticipatory and reactive components of postural control, highlighting how strategies evolve across distinct movement phases. Finally, the application of SPM allowed for a temporally continuous evaluation of COP dynamics across the task, avoiding oversimplification that arises from discrete-point measures and enabling the detection of subtle group differences over time. From a clinical perspective, these strengths extend beyond theoretical contributions. The CPT, combined with phase-specific and SPM-based analyses, may offer a sensitive tool for refining clinical assessments of postural control by capturing subtle immaturities that traditional static balance tests overlook. Moreover, identifying whether children rely on conservative, safety-first strategies versus proactive, feedforward adjustments can help tailor age-appropriate interventions. For instance, training that gradually increases task complexity could encourage children to adopt more efficient anticipatory strategies, while clinical monitoring of support use (e.g., leaning on objects) may serve as a marker of developmental delays or disorders such as DCD.

## Conclusion

Collectively, these findings suggest that although children approaching adolescence can produce basic APA patterns, they remain less adept at tailoring these responses to specific task demands and distributing them effectively across the movement sequence. This developmental gap is most evident in tasks requiring rapid and context-sensitive adjustments, reinforcing the value of phase-specific analyses to capture subtle but meaningful age-related differences in postural control.

## Data Availability

The raw data supporting the conclusions of this article will be made available by the authors, without undue reservation.
